# Socioeconomic Inequalities and Vaccine Uptake: An Umbrella Review Protocol

**DOI:** 10.3390/ijerph191811172

**Published:** 2022-09-06

**Authors:** Amber Sacre, Clare Bambra, Josephine M. Wildman, Katie Thomson, Sarah Sowden, Adam Todd

**Affiliations:** 1 Population Health Sciences Institute, Newcastle University, Newcastle NE1 4LP, UK; 2 National Institute for Health and Care Research (NIHR) Applied Research Collaboration (ARC) North East and North Cumbria (NENC), Newcastle NE3 3XT, UK; 3 School of Pharmacy, Newcastle University, Newcastle NE1 7RU, UK

**Keywords:** vaccine uptake, routine vaccinations, socioeconomic inequalities, evidence synthesis

## Abstract

The effectiveness of immunization is widely accepted: it can successfully improve health outcomes by reducing the morbidity and mortality associated with vaccine-preventable diseases. In the era of pandemics, there is a pressing need to identify and understand the factors associated with vaccine uptake amongst different socioeconomic groups. The knowledge generated from research in this area can be used to inform effective interventions aimed at increasing uptake. This umbrella systematic review aims to determine whether there is an association between socioeconomic inequalities and rate of vaccine uptake globally. Specifically, the study aims to determine whether an individual’s socioeconomic status, level of education, occupation, (un)-employment, or place of residence affects the uptake rate of routine vaccines. The following databases will be searched from 2011 to the present day: Medline (Ovid), Embase (Ovid), CINAHL (EBSCO), Cochrane CENTRAL, Science Citation Index (Web of Science), DARE, SCOPUS (Elsevier), and ASSIA (ProQuest). Systematic reviews will be either included or excluded based on a priori established eligibility criteria. The relevant data will then be extracted, quality appraised, and narratively synthesised. The synthesis will be guided by the theoretical framework developed for this review. The Preferred Reporting Items for Systematic Reviews and Meta-Analyses Equity extension (PRISMA-E) guidance will be followed. This protocol has been registered on PROSPERO, ID: CRD42022334223.

## 1. Introduction

In the “era of pandemics” [1], there is an increasing need to identify and understand the barriers which prevent or reduce the uptake of vaccines. Vaccines are regarded as one of the most cost-effective public health interventions and can improve health outcomes by reducing morbidity and mortality associated with vaccine-preventable diseases [2]. Due to the effectiveness of immunisation programmes, vaccination has been identified by the World Health Organisation (WHO) as an “indisputable human right” and should be accessible to the entire global population [3]. However, access to vaccinations is far from universal; for example, it is estimated that, in 2020, 23 million children under the age of one did not receive their basic vaccinations [3]. There are many reasons for people to remain unvaccinated, many of which can vary across time (recently implemented vaccines versus long-established vaccines) and space (contextual differences between high-income and low-income countries) [2,4]. More generally, discussions of healthcare “uptake” explore the presence of physical and non-physical barriers that contribute to whether an individual receives a vaccine or not [2]. These barriers to access are reproduced by the individual, healthcare service providers, and wider societal inequalities [5].

**Figure 1:**
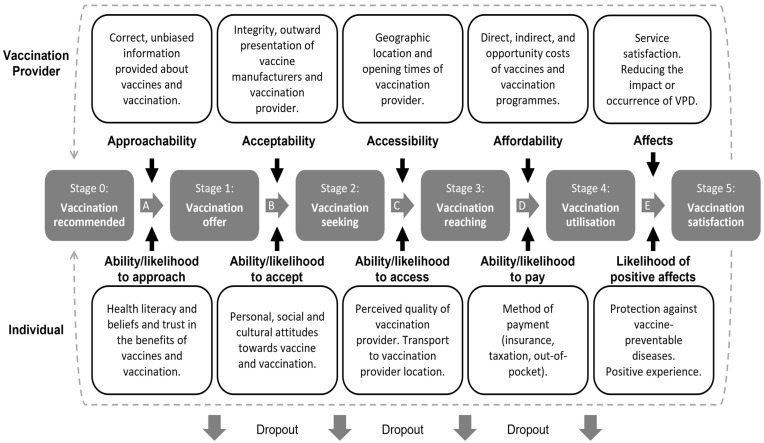
Illustrates the process of access to vaccinations. Adapted from Levesque et al.’s patient-centred access to healthcare framework [7].

The Preferred Reporting Items for Systematic Reviews and Meta-Analyses Equity extension (PRISMA-E) guidance, considered as best practice for conducting and reporting reviews that have an equity focus, will be utilised in this umbrella review [6]. One key element of this checklist is the inclusion of a framework to assist in the conceptualisation of a given issue [6]. Figure 1 details a framework outlining the stages of accessing vaccinations. The framework has been adapted from Levesque et al.’s patient-centred access to healthcare framework [7] which will be used in this work to conceptualise the factors which affect access to vaccinations.

The framework illustrates a patient-centred approach to accessing vaccination. Each of the middle boxes represents a key stage in the process. The terms “approachability”, “acceptability”, “accessibility”, “affordability”, and “affects” refer to the considerations of the vaccination provider. On the other hand, the ability/likelihood to “approach”, “accept”, “access”, “pay” and the likelihood of “positive affects” describe the concerns of the individual. Each of these terms can represent a barrier that prevents progress to the next stage and can be viewed as mediators. It should be emphasised that the process of vaccination does not end with the administration of the vaccine; the overall experience could affect an individual’s likelihood to reengage with the service. This can be a significant problem in the context of multi-dose vaccination schedules where adherence is crucial for maximising protection. Indeed, if the mediators are not adequately addressed, individuals can drop out of the process before reaching the next stage. Findings from the proposed umbrella review will be used to further adapt the framework to illustrate the role of SE determinants in access to vaccination.

As demonstrated, there are multiple influences which can impact access to vaccination, but how do these influencers interact with socioeconomic (SE) inequalities? It is widely accepted that SE factors impact access to healthcare across the globe [8,9,10]. Often, those who are disadvantaged face more barriers when attempting to access healthcare services [10]; they are more likely to delay medical treatment [9] and experience longer waiting times for required services [8]. Socioeconomic status (SES) is often employed in discussions of healthcare uptake as a measure of someone’s socioeconomic position in society. SES can be operationalised in a variety of ways, at both the individual and area-level. Some common measures are: annual income, occupational classification, level of education, deprivation, or a socially constructed notion of an individual’s hierarchical position in society (e.g., socioeconomic status) [5]. This review is concerned with inequalities that can be considered avoidable and unfair [5,11,12]. It will utilise the four elements of the PROGRESS framework which was created to conceptualise barriers to equitable healthcare access [13]: education, occupation, socioeconomic status (income), and place of residence (area-level deprivation).

There is conflicting evidence as to whether vaccine uptake is affected by SES. COVID-19 cumulative uptake statistics from England found differing rates according to indices of multiple deprivation quintiles; 19.2% of men categorised as quintile 1 (most deprived) had not received any COVID-19 vaccines as of May 2022, compared to only 6.8% of men in quintile 5 (least deprived) [14]. However, a systematic review published by Larson et al. in 2014 explored hesitancy towards childhood vaccines from a global perspective. The research provides a useful overview of the vaccine uptake discourse and the complexities associated with it. There are, however, a number of challenges that affect this work: for example, income and SES are used interchangeably, making it difficult to identify the impact of each measure and understand how socioeconomic status is operationalised [4]. Table 1 provides a summary of their findings. Both high income/SES/education and low income/SES/education are significant as promoters and barriers to vaccine uptake.

Another systematic review exploring the uptake of the Measles-Mumps-Rubella (MMR) and Diphtheria-Tetanus-Pertussis-containing (DTaP) vaccines amongst infants and pre-school children in Europe and Australia has also been conducted, [44]. This review concluded that socioeconomic differences in uptake were often greater in a specific circumstance, namely, in non-hierarchical primary care organisations without well-baby clinics [44]. Other systematic reviews suggest that socioeconomic inequality in vaccine uptake could be a proxy for racial and ethnic inequalities, especially when considering the human papillomavirus (HPV) vaccine [45,46]. Ethnic minority individuals are more likely to experience deprivation than their white counterparts, which is therefore intrinsically linked to SE factors. Overall, existing evidence contains significant variations in study characteristics and the subsequent findings.

Systematic reviews and meta-analyses, in isolation, often cannot adequately explain why a relationship has occurred, but they can aid the development of further hypotheses to investigate the “why” aspect. One means of doing so is to explore mechanisms and pathways which may cause or contribute to the occurrence of an association. A critique of existing evidence on SE health and healthcare inequalities in general is its infrequent exploration of the mechanisms and pathways by which SES may cause its impact [47]. The mechanisms by which SE status may influence vaccine uptake are also likely to differ between countries. For instance, in the context of the UK, vaccination is provided by a national healthcare system funded through general taxation. Subsequently, there is no direct economic consequence of utilisation. In contrast, the United States healthcare system is primarily market-driven, and access to vaccination is chiefly reliant on insurance [48]. Fisher et al. found that women in the United States without health insurance were less likely to be vaccinated against HPV [49]. Health insurance is either funded through income or provided by employers. Those without insurance must use out-of-pocket payments [50] to fund vaccination unless they are eligible for government assistance. Understanding both the determinants and mechanisms of SE inequalities is crucial for designing effective interventions to increase vaccine uptake.

The aim of this umbrella review is to ascertain whether there are socioeconomic inequalities in vaccine uptake. If associations are found, the review, where possible, will identify mechanisms and pathways which may contribute to inequalities in vaccine uptake. To our current knowledge, an umbrella review exploring this topic in sufficient detail has not been published. This review will simultaneously identify areas where further research is required and identify sub-populations at risk for low vaccine uptake.

## 2. Methods

Umbrella reviews represent the highest level of evidence synthesis. Occasionally, the terms “overview of reviews” or “review of reviews” are used instead of “umbrella review”, but there are distinct differences between them [51,52,53]. An umbrella review “refers to [a] review compiling evidence from multiple reviews into one accessible usable document” [52]. It is appropriate to conduct this type of systematic review because more than one intervention (vaccine) is being explored [52].

### 2.1. Research Questions

The research questions for the umbrella review are:

- **Primary:** Are there socioeconomic inequalities in vaccine uptake?
  If so, which vaccines, countries, and measures of socioeconomic status are affected?
- **Secondary:** Are any potentially impactful mechanisms or pathways of socioeconomic inequalities in vaccine uptake identified?
  If so, what mechanisms or pathways are identified?

### 2.2. Inclusion Criteria

- **Population:** All countries, and demographical and social groups, will be eligible for inclusion.
- **Interventions:** The intervention, or phenomena of interest, are WHO universally recommended routine vaccinations. All WHO recommended routine vaccines will be considered (see Table 2 for more information on what constitutes a routine vaccination), including influenza and COVID-19, to account for reviews published in response to the Coronavirus pandemic.
- **Comparison:** Systematic reviews will be included irrespective of whether their primary studies had controls or not. Control groups may include randomised or matched designs.
- **Outcomes:** Variation in the rate or proportion of a target population which have been vaccinated, according to socioeconomic determinants. The SE determinants will be: the level of education, occupational classification, measures of area-level deprivation (e.g., the English Indices of Multiple Deprivation [54]), and income.
- **Study Design:** Only systematic reviews or studies which attempt to synthesise quantitative or qualitative primary studies will be included. The quantitative reviews do not have to include a meta-analysis.

A systematic review is classified as such if it meets four of the following criteria outlined by the Database of Abstracts of Reviews of Effects (DARE) [55]:

1. Were inclusion/exclusion criteria reported?
2. Was the search adequate?
3. Were the included studies synthesised?
4. Was the quality of the included studies assessed?
5. Are sufficient details about the individual included studies presented?

A date restriction of 2011 to the present was applied. The WHO published the ‘Global Vaccine Action Plan 2011–2020’ report which provides updated guidance on improving vaccine coverage and uptake [56]. This ensures the findings from the included systematic reviews are relevant in 2022. The full inclusion and exclusion criteria can be seen in Table 2.

### 2.3. Information Sources and Search Strategy

The following databases will be searched from 2011 to the present day (host sites given in parentheses): Medline (Ovid), Embase (Ovid), CINAHL (EBSCO), Cochrane CENTRAL, Science Citation Index (Web of Science), Database of Abstract Reviews of Effects, SCOPUS (Elsevier), and Applied Social Sciences Index and Abstracts (ASSIA) (ProQuest). Grey literature searching will be conducted using the WHO repositories and PROSPERO. These sources will be searched from the 9 February 2022 to 9 September 2022. Forward and backwards citation of included systematic reviews and key paper searching will be conducted to ensure all relevant reviews are identified. Both free text and subject headings will be used in the searches and combined with the appropriate Boolean operators. The four main groupings of the search terms are: population, intervention, outcome, and study design. The search strategy can be seen in the supplementary material (Annex S2) and will be adapted to each of the databases.

### 2.4. Screening and Selection

The publications identified by the inclusion and exclusion criteria will be downloaded into Rayyan [58], where the results of all searches will be collated and de-duplicated. Using the Rayyan software [58], the main reviewer (AS) will blindly assign publications to a second reviewer for checking. The articles will be pre-screened by the main reviewer (AS) by their title and abstract and 10% will also have their title and abstract double-screened by a second, independent reviewer (JMW). After the pre-screening stage, the remaining articles will have their full text examined by reviewer one (AS) with consideration of the inclusion and exclusion criteria. Either the second (JMW) or a third (KT) reviewer will then independently check 10% of the publications deemed potentially eligible for inclusion. If there is any debate over the inclusion or exclusion of a systematic review at any stage of the pre- or screening process, a majority decision will be made between the three reviewers. The screening process will be summarised in a PRISMA flow diagram and included in the final publication [59].

### 2.5. Data Extraction

Both quantitative and qualitative data will be extracted from the included reviews and summarised in a table (see supplementary material Annex S4). Reviewer one (AS) will perform the data extraction. The following information will be obtained from the systematic reviews: bibliographical and characteristic details (author, year of publication, title, DOI, study type, and number of included studies); any information relating to PICOS (geographical location, vaccine/s, measure of socioeconomic inequality); name and date of databases searches; inclusion and exclusion criteria; method of data synthesis; the main findings/conclusions relevant to socioeconomic inequalities in vaccine uptake; any mention of mechanisms relevant to education, occupation, income, and area-level deprivation; and results of the risk of bias assessment. Additionally, the primary studies included within the reviews will have the following information extracted: authors, year of publication, title, and study design. Either reviewer two (JMW) or reviewer three (KT) will double-check the extraction form to ensure the data are correct.

### 2.6. Quality Appraisal

Each of the included systematic reviews will be assessed for their quality using a critical appraisal tool. There are many different tools available, but this review will utilise the AMSTAR 2 checklist because it can be used to assess both randomised and non-randomised control trials [60]. Studies will be categorised as high, moderate, low, or critically low-quality based on the proportion of criteria which have been met. The results from the checklist will be summarised in a table and included in the final publication. The quality appraisal process will be undertaken by the first reviewer (AS) and checked by the second (JMW) or third (KT) reviewer. Studies determined to be of poor quality will still be included in the review, but their conclusions will be discussed with caution. It must be acknowledged that quality appraisal is subjective, but bias is reduced when using a checklist for consistency.

### 2.7. Dealing with Overlap

There is a high-risk of overlap in primary studies when conducting an umbrella review [61]. A citation matrix will be used to ascertain the frequency at which each primary study occurs across the included systematic reviews: see supplementary material Annex S6 [62]. There are several ways of addressing issues of overlap, but the chosen method must be decided based on the nature of the overlap [61], such as reporting the corrected coverage area (CCA). These decisions will be discussed with the second (JW) and third reviewers (KT) if significant overlap occurs.

## 3. Results

### 3.1. Synthesis

Following successful screening, extraction, and quality appraisal, the data will be narratively synthesised. A narrative approach to synthesis is most appropriate when considering an array of different contexts and interventions across the included systematic reviews [62]. The synthesis will be guided by the framework produced for this review, which can be seen in Figure 1, and the Synthesis Without Meta-analysis recommendations [63]. The following information for each of the included reviews will be summarised in a table (see supplementary material Annex S7): authors and year; country/countries where the study is set; vaccine/s; measures of socioeconomic inequality (education, occupation, income, and area-level deprivation); key findings related to socioeconomic inequalities in vaccine uptake; a description of any mechanisms identified; types of mechanisms (according to the framework); and AMSTAR 2 rating. Next, the similarities and differences between the included reviews will be briefly described. Specifically, whether there are any commonalities between reviews which claim SE determinants do have an impact on vaccine uptake. Throughout the synthesis, the results of the quality appraisal will be considered to ensure the conclusions of low-quality systematic reviews do not skew the umbrella review findings. Any mechanisms or pathways identified will be mapped to the framework (see Figure 1), which will then be adapted to incorporate the impact of socioeconomic determinants on the access to vaccination processes.

### 3.2. Pilot Search

A pilot search was conducted using Medline (Ovid) on 09/02/2022 to test the search strategy. This was done in two stages. Eight systematic reviews were selected to be used as key indicator papers in the pilot searches [44,46,49,64,65,66,67,68]. A search string developed by BMJ Best Practice to retrieve systematic reviews was implemented for the study design element of the strategy [69].

- **Pilot search 1** consisted of the study design, population, and intervention elements of the search strategy. The search returned 2087 results, of which all nine key papers were included (see supplementary material Annex S8).
- **Pilot search 2** consisted of the study design, population, intervention, and outcome elements of the search strategy. The searched returned 1090 results, of which all nine key papers were included (see supplementary material Annex S9).

A decision was made to use the approach of pilot search 2. The results are significantly reduced in the second search, but still returned all nine key papers.

## 4. Discussion

Despite the proven effectiveness of vaccination, there are many individuals who remain unvaccinated [3]. Understanding the complexity of vaccine uptake in the “era of pandemics” is crucial [1]. The current rate of routine vaccine uptake needs to be safeguarded and, where possible, improved. However, the resurfacing of vaccine hesitancy sentiments surrounding the COVID-19 vaccine has the potential to affect uptake of other vaccines. If vaccine rates begin to decrease, the occurrence of vaccine-preventable diseases will subsequently increase.

Unfair and avoidable inequalities potentially affect the distribution of vaccine uptake across different SE groups; however, the existing evidence is complex and variable. This umbrella review will provide a summary of systematic reviews that explore the impact of SES on the uptake of routine vaccination. It will identify the vaccines, countries, and measures of SES that affect the distribution of uptake. There will be an exploration of the similarities and differences between reviews that claim an association exists. Any potentially influential mechanisms or pathways that lead to SES being found as influential will also be summarised. This umbrella review exists as one component of a wider investigation into SES inequalities in vaccine uptake. The findings will provide a direction for the remaining components, supported by data from the highest level of evidence synthesis. Furthermore, by extracting information on any mechanisms and pathways, it can be used to aid future research into effective interventions to reduce the impact and prevalence of SE factors in the discussion of vaccine uptake. Identification and exploration of these mechanisms and pathways will minimise the risk of developing an intervention that perpetuates SE inequalities [70].

One potential limitation of this umbrella review is the absence of a population restriction and a broad inclusion criterion for the intervention. Subsequently, all countries and routine vaccinations are eligible. This comprehensive approach can make it difficult to identify whether there are any true commonalities due to the differing contexts. Additionally, it is possible that some recent primary studies will not be included because they have not yet been synthesised into a systematic review [52]. However, it will follow the PRISMA-E guidance and utilise the AMSTAR 2 checklist for quality-appraisal. Using these well-established tools will ensure that the research is methodologically robust.

## 5. Conclusions

This umbrella review will make a positive and noteworthy contribution to the discourse of SE inequalities in vaccine uptake by providing an overview of the existing evidence and simultaneously identifying gaps that require further exploration. It will help identify which groups need support accessing vaccines in order to reduce inequalities in vaccine-preventable morbidity and mortality.

## Figures and Tables

**Table 1:** Summary of Larson et al.’s systematic review findings [4].

	Barrier	Promoter
Low income/SES	USA [15] Nigeria [16]	Nigeria [17] Bangladesh [18]
High income/SES	USA [19]	Burkina Faso [20,21] India [22] Bangladesh [18]
Low education	Nigeria [16,17,23,24] India [25,26] China [27] Kyrgyzstan [28] USA [29] DR Congo [30]	USA [31]
High education	China [32] Lebanon [33] Israel [34] Bangladesh [18] USA [19] DR Congo [30]	India [22,25,35,36,37,38] Greece [39] The Netherlands [40] Nigeria [41] Pakistan [42,43]

**Table 2:** Inclusion and exclusion criteria.

Inclusion	Exclusion
Access to the full text.	
Reviews published after 2011–present day. Any language (interpreters will be sourced if required).	
**Inclusion:** Population All countries. Normal/general populations. Reviews which focus on demographical sub-populations.	**Exclusion:** Population Reviews which focus on an occupational sub-population (such as health care workers). Reviews which focus on clinically at-risk populations (such as diabetics and pregnant women).
**Inclusion:** Intervention Reviews which focus on vaccine **uptake**. WHO-recommended routine vaccinations universally or worldwide [57]. BCG (Tuberculosis), Hepatitis B, Polio, DTP-containing vaccine (Diphtheria, Tetanus and Pertussis), Haemophilus influenzae type b, Pneumococcal (conjugate), Rotavirus, Measles, Rubella, and HPV (Human papillomavirus). Influenza and COVID-19 vaccinations.	**Exclusion:** Intervention Reviews which focus on interventions to improve vaccine uptake. WHO vaccine recommendations for certain regions (Japanese Encephalitis, Yellow Fever, Tick-Borne Encephalitis) [57]. WHO vaccine recommendations for some high-risk populations (Typhoid, Cholera, Meningococcal, Hepatitis A, Rabies, and Dengue) [57]. WHO vaccine recommendations for immunisation programs with certain characteristics (Mumps and Varicella) [57].
**Inclusion:** Comparison Control groups may include randomised or matched designs. Other comparison groups will also be considered, such as preintervention and postintervention or alternative intervention comparisons.	**Exclusions:** Comparison No exclusions.
**Inclusion:** Outcome Reviews focusing on socioeconomic inequalities, specifically education, occupation, income, and area-level deprivation. Reviews which report the rate or proportion of a target population that have been vaccinated. Reviews which report initiation and/or completion of vaccination programmes.	**Exclusion:** Outcome Vaccine uptake targets or estimation models.
**Inclusion:** Study Design Must be a systematic review, as defined by the DARE criteria [55]. Must synthesise primary empirical studies.	**Exclusion:** Study Design Studies which state they are reviews but do not meet four or more of the DARE criteria [55] or are a primary study or a conference paper. Mixed reviews where the relevant information cannot be separated from the irrelevant. Reviews which attempt to synthesize secondary data.

## Data Availability

Not applicable.

## Supplementary Materials

The following supporting information can be downloaded at: https://www.mdpi.com/article/10.3390/ijerph191811172/s1, File S1: Annex S1: PRISMA-E checklist; Annex S2: search strategy; Annex S3: PRISMA-flow template; Annex S4: data extraction tables; Annex S5: AMSTAR-2 checklist; Annex S6: citation matrix; Annex S7: summary tables; Annex S8: pilot search 1; Annex S9: pilot search 2; Annex S10: BMJ search design filters.

## References

[1] UN Environmental Programme (2020). Escaping the ‘Era of Pandemics’: Experts Warn Worse Crises to Come Options Offered to Reduce Risk.

[2] SAGE Working Group (2014). Report of the SAGE Working Group of Vaccine Hesitancy. https://www.asset-scienceinsociety.eu/sites/default/files/sage_working_group_revised_report_vaccine_hesitancy.pdf.

[3] Vaccines and Immunization. https://www.who.int/health-topics/vaccines-and-immunization#tab=tab_1.

[4] Larson H.J., Jarrett C., Eckersberger E., Smith D.M.D., Paterson P. (2014). Understanding vaccine hesitancy around vaccines and vaccination from a global perspective: A systematic review of published literature, 2007–2012. Vaccine.

[5] Graham H. (2007). Unequal Lives: Health and Socioeconomic Inequalities.

[6] Welch V., Petticrew M., Tugwell P., White H., PRISMA-Equity Bellagio Group (2012). PRISMA-Equity 2012 extension: Reporting guidelines for systematic reviews with a focus on health equity. PLoS Med..

[7] Levesque J.F., Harris M.F., Russell G. (2013). Patient-centred access to health care: Conceptualising access at the interface of health systems and populations. Int. J. Equity Health.

[8] Moscelli G., Siciliani L., Gutacker N., Cookson R. (2018). Socioeconomic inequality of access to healthcare: Does choice explain the gradient?. J. Health Econ..

[9] Gordon T., Booysen F., Mbonigaba J. (2020). Socio-economic inequalities in the multiple dimensions of access to healthcare: The case of South Africa. BMC Public Health.

[10] Walters S., Suhrcke M. (2005). Socioeconomic Inequalities in Health and Health Care Access in Central and Eastern Europe and the CIS: A Review of the Recent Literature.

[11] O’Neill J., Tabish H., Welch V., Petticrew M., Pottie K., Clarke M., Evans T., Pardo Pardo J., Waters E., White H. (2014). Applying an equity lens to interventions: Using PROGRESS ensures consideration of socially stratifying factors to illuminate inequities in health. J. Clin. Epidemiol..

[12] Norman C., Wildman J.M., Sowden S. (2021). COVID-19 at the Deep End: A Qualitative Interview Study of Primary Care Staff Working in the Most Deprived Areas of England during the COVID-19 Pandemic. Int. J. Environ. Res. Public Health.

[13] PROGRESS-Plus. https://methods.cochrane.org/equity/projects/evidence-equity/progress-plus.

[14] (2022). Coronavirus and Vaccination Rates in People Aged 18 Years or Over by Socio-Demographic Characteristic and Region, England.

[15] Wu A.C., Wisler-Sher D.J., Griswold K., Colson E., Shapiro E.D., Holmboe E.S. (2008). Postpartum mothers’ attitudes, knowledge, and trust regarding vaccination. Matern. Child Health J..

[16] Antai D. (2009). Faith and child survival: The role of religion in childhood immunization in Nigeria. J. Biosoc. Sci..

[17] Antai D. (2012). Gender inequities, relationship power, and childhood immunization uptake in Nigeria: A population-based cross-sectional study. Int. J. Infect. Dis..

[18] Rahman M., Obaida-Nasrin S. (2010). Factors affecting acceptance of complete immunization coverage of children under five years in rural Bangladesh. Salud Publica Mex..

[19] Wei F., Mullooly J.P., Goodman M., McCarty M.C., Hanson A.M., Crane B., Nordin J.D. (2009). Identification and characteristics of vaccine refusers. BMC Pediatr..

[20] Sanou A., Simboro S., Kouyaté B., Dugas M., Graham J., Bibeau G. (2009). Assessment of factors associated with complete immunization coverage in children aged 12-23 months: A cross-sectional study in Nouna district, Burkina Faso. BMC Int. Health Hum. Rights.

[21] Sia D., Fournier P., Kobiane J.F., Sondo B.K. (2009). Rates of coverage and determinants of complete vaccination of children in rural areas of Burkina Faso (1998–2003). BMC Public Health.

[22] Patra N. (2012). A probe into the ways to stimulate immunisation in India: Findings from National Family Health Survey-III. IJCP.

[23] Babalola S. (2011). Maternal reasons for non-immunisation and partial immunisation in northern Nigeria. J. Paediatr. Child Health.

[24] Oladokun R., Adedokun B., Lawoyin T. (2010). Children not receiving adequate immunization in Ibadan, Nigeria: What reasons and beliefs do their mothers have?. Niger. J. Clin. Pract..

[25] Kumar D., Aggarwal A., Gomber S. (2010). Immunization status of children admitted to a tertiary-care hospital of north India: Reasons for partial immunization or non-immunization. J. Health Popul. Nutr..

[26] Patel T., Pandit N. (2011). Why infants miss vaccination during routine immunization sessions? Study in a rural area of Anand district, Gujarat. Indian J. Public Health.

[27] Wang Y.Y., Wang Y., Zhang J.X., Kang C.Y., Duan P. (2007). Status of mother’s KAP on child immunization in minority areas, Guizhou Province. Beijing Da Xue Xue Bao Yi Xue Ban.

[28] Akmatov M.K., Mikolajczyk R.T., Kretzschmar M., Krämer A. (2009). Attitudes and beliefs of parents about childhood vaccinations in post-soviet countries: The example of Kyrgyzstan. Pediatr. Infect Dis. J..

[29] Stockwell M.S., Irigoyen M., Martinez R.A., Findley S. (2011). How Parents’ Negative Experiences at Immunization Visits Affect Child Immunization Status in a Community in New York City. Public Health Rep..

[30] Mapatano M., Kayembe K., Piripiri L., Nyandwe K. (2008). Immunisation-related knowledge, attitudes and practices of mothers in Kinshasa, Democratic Republic of the Congo. SA Fam. Pract..

[31] Kim S.S., Frimpong J.A., Rivers P.A., Kronenfeld J.J. (2007). Effects of Maternal and Provider Characteristics on Up-to-Date Immunization Status of Children Aged 19 to 35 Months. Am. J. Public Health.

[32] Zhang S., Yin Z., Suraratdecha C., Liu X., Li Y., Hills S., Zhang K., Chen Y., Liang X. (2011). Knowledge, attitudes and practices of caregivers regarding Japanese encephalitis in Shaanxi Province, China. Public Health.

[33] Sinno D.D., Shoaib H.A., Musharrafieh U.M., Hamadeh G.N. (2009). Prevalence and predictors of immunization in a health insurance plan in a developing country. Pediatr. Int..

[34] Muhsen K., El-Hai R.A., Amit-Aharon A., Nehama H., Gondia M., Davidovitch N., Goren S., Cohen D. (2012). Risk factors of underutilization of childhood immunizations in ultraorthodox Jewish communities in Israel despite high access to health care services. Vaccine.

[35] Phukan R.K., Barman M.P., Mahanta J. (2009). Factors Associated with immunization coverage of children in Assam, India: Over the first year of life. J. Trop Pediatr..

[36] Chhabra P., Nair P., Gupta A., Sandhir M., Kannan A.T. (2007). Immunization in urbanized villages of Delhi. Indian J. Pediatr..

[37] Rammohan A., Awofeso N., Fernandez R.C. (2012). Paternal education status significantly influences infants’ measles vaccination uptake, independent of maternal education status. BMC Public Health.

[38] Vikram K., Vanneman R., Desai S. (2012). Linkages between maternal education and childhood immunization in India. Soc. Sci Med..

[39] Danis K., Georgakopoulou T., Stavrou T., Laggas D., Panagiotopoulos T. (2012). Socioeconomic factors play a more important role in childhood vaccination coverage than parental perceptions: A cross-sectional study in Greece. Vaccine.

[40] Uwemedimo O.T., Findley S.E., Andres R., Irigoyen M., Stockwell M.S. (2012). Determinants of Influenza Vaccination Among Young Children in an Inner-City Community. J. Community Health.

[41] Oladokun R.E., Lawoyin T.O., Adedokun B.O. (2009). Immunization status and its determinants among children of female traders in Ibadan, South-Western Nigeria. Afr. J. Med. Med. Sci..

[42] Mitchell S., Andersson N., Ansari N.M., Omer K., Soberanis J.L., Cockcroft A. (2009). Equity and vaccine uptake: A cross-sectional study of measles vaccination in Lasbela District, Pakistan. BMC Int. Health Hum. Rights.

[43] Siddiqi N., Siddiqi A.E.A., Nisar N., Khan A. (2010). Mothers’ knowledge about EPI and its relation with age-appropriate vaccination of infants in peri-urban Karachi. J. Pak. Med. Assoc..

[44] Arat A., Burström B., Östberg V., Hjern A. (2019). Social inequities in vaccination coverage among infants and pre-school children in Europe and Australia—A systematic review. BMC Public Health.

[45] Jeudin P., Liveright E., del Carmen M.G., Perkins R.B. (2013). Race, ethnicity and income as factors for HPV vaccine acceptance and use. Hum. Vaccin Immunother..

[46] Fernández de Casadevante V., Gil Cuesta J., Cantarero-Arévalo L. (2015). Determinants in the Uptake of the Human Papillomavirus Vaccine: A Systematic Review Based on European Studies. Front. Oncol..

[47] Glymour M.M., Avendano M., Kawachi I., Berkman L., Kawachi I., Glymour M. (2015). Socioeconomic Status and Health. Social Epidemiology.

[48] Sun X. (2019). World Health Systems.

[49] Fisher H., Trotter C.L., Audrey S., MacDonald-Wallis K., Hickman M. (2013). Inequalities in the uptake of human papillomavirus vaccination: A systematic review and meta-analysis. Int. J. Epidemiol..

[50] Glossary: Out-of-of-Pocket Expenditure on Healthcare. https://ec.europa.eu/eurostat/statistics-explained/index.php?title=Glossary:Out-of-pocket_expenditure_on_healthcare#:~:text=Household%20out%2Dof%2Dpocket%20payment,the%20use%20of%20the%20services.

[51] Martinic K.M., Pieper D., Glatt A., Puljak L. (2019). Definition of a systematic review used in overviews of systematic reviews, meta-epidemiological studies and textbooks. BMC Med. Res. Methodol..

[52] Grant M.J., Booth A. (2009). A typology of reviews: An analysis of 14 review types and associated methodologies. Health Info. Libr. J..

[53] Tsagris M., Fragkos K.C., Biondi-Zoccai G. (2016). Umbrella Reviews, Overviews of Reviews, and Meta-epidemiologic Studies: Similarities and Differences. Umbrella Reviews: Evidence Synthesis with Overviews of Reviews and Meta-Epidemiologic Studies.

[54] English Indices of Deprivation. https://www.gov.uk/government/statistics/english-indices-of-deprivation-2019.

[55] Database of Abstracts of Reviews of Effects (DARE): Quality Assessed Reviews. https://www.ncbi.nlm.nih.gov/books/NBK285222/.

[56] World Health Organization (2013). Global Vaccine Action Plan. 2011–2020.

[57] World Health Organization (2021). Table 1: Summary of WHO Position Papers—Recommendations for Routine Immunization.

[58] (2016). Rayyan.

[59] Page M.J., McKenzie J.E., Bossuyt P.M., Boutron I., Hoffmann T.C., Mulrow C.D., Shamseer L., Tetzlaff J.M., Akl E.A., Brennan S.E. (2021). The PRISMA 2020 statement: An updated guideline for reporting systematic reviews. BMJ.

[60] Shea B.J., Reeves B.C., Wells G., Thuku M., Hamel C., Moran J., Moher D., Tugwell P., Welch V., Kristjansson E. (2017). AMSTAR 2: A critical appraisal tool for systematic reviews that include randomised or non-randomised studies of healthcare interventions, or both. BMJ.

[61] Lunny C., Pieper D., Thabet P., Kanji S. (2021). Managing overlap of primary study results across systematic reviews: Practical considerations for authors of overviews of reviews. BMC Med. Res. Methodol..

[62] Pollock M., Fernandes R., Becker L., Pieper D., Hartling L., Higgins J., Thomas J., Chandler J., Cumpston M., Li T., Page M.J., Welch V.A. (2022). Chapter V: Overviews of Reviews. Cochrane Handbook for Systematic Reviews of Interventions, 6.3.

[63] Campbell M., McKenzie J., Sowden A., Katikireddi S., Brennan S., Ellis S., Hartmann-Boyce J., Ryan R., Shepperd S., Thomas J. (2020). Synthesis without meta-analysis (SWiM) in systematic reviews: Reporting. BMJ.

[64] Crocker-Buque T., Mindra G., Duncan R., Mounier-Jack S. (2017). Immunization, urbanization and slums—A systematic review of factors and interventions. BMC Public Health.

[65] Gallagher K.E., Kadokura E., Eckert L.O., Miyake S., Mounier-Jack S., Aldea M., Ross D.A., Watson-Jones D. (2016). Factors influencing completion of multi-dose vaccine schedules in adolescents: A systematic review. BMC Public Health.

[66] Tabacchi G., Costantino C., Napoli G., Marchese V., Cracchiolo M., Casuccio A., Vitale F., The Esculapio Working Group (2016). Determinants of European parents’ decision on the vaccination of their children against measles, mumps and rubella: A systematic review and meta-analysis. Hum. Vaccin Immunother..

[67] Forshaw J., Gerver S.M., Gill M., Cooper E., Manikam L., Ward H. (2017). The global effect of maternal education on complete childhood vaccination: A systematic review and meta-analysis. BMC Infect Dis..

[68] Bocquier A., Wardm J., Raude J., Peretti-Watel P., Verger P. (2017). Socioeconomic differences in childhood vaccination in developed countries: A systematic review of quantitative studies. Expert Rev. Vaccine.

[69] Study Design Search Filters. https://bestpractice.bmj.com/info/toolkit/learn-ebm/study-design-search-filters/.

[70] Todd A., Bambra C. (2021). Learning from past mistakes? The COVID-19 vaccine and the inverse equity hypothesis. Eur. J. Public Health.

