# Supplementary material for: Socioeconomic Inequalities and Vaccine Uptake: An Umbrella Review Protocol
Source: Int J Environ Res Public Health. 2022 Sep 6;19(18):11172. doi: 10.3390/ijerph191811172 (PMC9517548; doi:10.3390/ijerph191811172)
Supplement: Supplementary file 1 [file ijerph-19-11172-s001.zip › ijerph-1864037-supplementary.pdf]

File S1

Annex S1: PRISMA-E checklist [4].

Checklist of Items for Reporting Equity-Focused Systematic Reviews				
Section	Item	Standard PRISMA Item	Extension for Equity-Focused Reviews	Pg #
Title				
Title	1	Identify the report as a systematic review, meta-analysis, or both.	Identify equity as a focus of the review, if relevant, using the term equity	
Abstract				
Structured summary	2	2. Provide a structured summary including, as applicable: background; objectives; data sources; study eligibility criteria, participants, and interventions; study appraisal and synthesis methods; results; limitations; conclusions and implications of key findings; systematic review registration number.	State research question(s) related to health equity.	
	2A		Present results of health equity analyses (e.g. subgroup analyses or meta-regression).	
	2B		Describe extent and limits of applicability to disadvantaged populations of interest.	
Introduction				
Rationale	3	Describe the rationale for the review in the context of what is already known.	Describe assumptions about mechanism(s) by which the intervention is assumed to have an impact on health equity.	
	3A		Provide the logic model/analytical framework, if done, to show the pathways through which the intervention is assumed to affect health equity and how it was developed.	
Objectives	4	Provide an explicit statement of questions being addressed with reference to participants, interventions, comparisons, outcomes, and study design (PICOS).	Describe how disadvantage was defined if used as criterion in the review (e.g. for selecting studies, conducting analyses or judging applicability).	
	4A		State the research questions being addressed with reference to health equity	
Methods				
Protocol and registration	5	Indicate if a review protocol exists, if and where it can be accessed (e.g., Web address), and, if available, provide registration information including registration number.		
Eligibility criteria	6	6. Specify study characteristics (e.g., PICOS, length of follow-up) and report characteristics (e.g., years considered, language, publication status) used as criteria for eligibility, giving rationale.	Describe the rationale for including particular study designs related to equity research questions.	

	6A		Describe the rationale for including the outcomes - e.g. how these are relevant to reducing inequity.	
Information sources	7	Describe all information sources (e.g., databases with dates of coverage, contact with study authors to identify additional studies) in the search and date last searched.	Describe information sources (e.g. health, non-health, and grey literature sources) that were searched that are of specific relevance to address the equity questions of the review.	
Search	8	Present full electronic search strategy for at least one database, including any limits used, such that it could be repeated.	Describe the broad search strategy and terms used to address equity questions of the review.	
Study selection	9	State the process for selecting studies (i.e., screening, eligibility, included in systematic review, and, if applicable, included in the meta-analysis).		
Data collection process	10	Describe method of data extraction from reports (e.g., piloted forms, independently, in duplicate) and any processes for obtaining and confirming data from investigators.		
Data items	11	List and define all variables for which data were sought (e.g., PICOS, funding sources) and any assumptions and simplifications made.	List and define data items related to equity,where such data were sought (e.g. using PROGRESS-Plus or other criteria, context).	
Risk of bias in individual studies	12	Describe methods used for assessing risk of bias of individual studies (including specification of whether this was done at the study or outcome level), and how this information is to be used in any data synthesis.		
Summary measures	13	State the principal summary measures (e.g., risk ratio, difference in means).		
Synthesis of results	14	Describe the methods of handling data and combining results of studies, if done, including measures of consistency (e.g., I^2) for each meta-analysis.	Describe methods of synthesizing findings on health inequities (e.g. presenting both relative and absolute differences between groups).	
Risk of bias across studies	15	15. Specify any assessment of risk of bias that may affect the cumulative evidence (e.g., publication bias, selective reporting within studies).		
Additional analyses	16	Describe methods of additional analyses (e.g., sensitivity or subgroup analyses, meta-regression), if done, indicating which were pre-specified.	Describe methods of additional synthesis approaches related to equity questions, if done, indicating which were pre-specified	
Results				
Study selection	17	Give numbers of studies screened, assessed for eligibility, and included in the review, with reasons for exclusions at each stage, ideally with a flow diagram.		
Study characteristics	18	For each study, present characteristics for which data were extracted (e.g., study size, PICOS, follow-up period) and	Present the population characteristics that relate to the equity questions across the relevant PROGRESS-Plus or other factors of	

		provide the citations.	interest.	
Risk of bias within studies	19	Present data on risk of bias of each study and, if available, any outcome level assessment (see item 12).		
Results of individual studies	20	For all outcomes considered (benefits or harms), present, for each study: (a) simple summary data for each intervention group (b) effect estimates and confidence intervals, ideally with a forest plot.		
Synthesis of results	21	Present results of each meta-analysis done, including confidence intervals and measures of consistency.	Present the results of synthesizing findings on inequities (see 14).	
Risk of bias across studies	22	Present results of any assessment of risk of bias across studies (see Item 15).		
Additional analysis	23	Give results of additional analyses, if done (e.g., sensitivity or subgroup analyses, meta-regression [see Item 16]).	Give the results of additional synthesis approaches related to equity objectives, if done, (see 16).	
Discussion				
Summary of evidence	24	Summarize the main findings including the strength of evidence for each main outcome; consider their relevance to key groups (e.g., healthcare providers, users, and policy makers).		
Limitations	25	Discuss limitations at study and outcome level (e.g., risk of bias), and at review-level (e.g., incomplete retrieval of identified research, reporting bias).		
Conclusions	26	Provide a general interpretation of the results in the context of other evidence, and implications for future research.	Present extent and limits of applicability to disadvantaged populations of interest and describe the evidence and logic underlying those judgments.	
	26A		Provide implications for research, practice or policy related to equity where relevant (e.g. types of research needed to address unanswered questions).	
Funding				
Funding	27	Describe sources of funding for the systematic review and other support (e.g., supply of data); role of funders for the systematic review.		

Annex S2: Search strategy.

[Population]
Title, Abstract, Key words=
socioeconomic or socio-economic or sociodemographic or sep or ses or class or
education or lifelong learning or life-long learning or human capital or school* or literacy or academic achievement or
employ* or unemploy* or occupation* or job* or work or career* or vocation or economic activity or labour market activity or isco or
income or wealth or wage* or salar* or earning* or low-income or money or
(inequit* or inequalit* or unequal or equal* or equit* or depriv* or poverty or impoverished or disadvantage* or gradient or gap* or disparit* or difference*) adj3 economic

[Intervention]
AND
Title, Abstract, Key words=
vaccine* or immunize or immunise or injection* or jab* or inoculate or
(tb or tuberculosis or Hep B or Hepatitis B or diphtheria or tetanus or pertussis or whooping cough or hib or haemophilus or haemophilus influenzae type b or poliovirus or polio or poliomyelitis or pneumococcal or pneumococcus or rotavirus or measles or rubella or human papillomavirus or wart virus or influenza or flu or COVID-19 or COVID 19 or COVID19 or coronavirus or SARS-CoV-2 or SARS Cov 2 or severe acute respiratory syndrome) adj3 vaccine* or
(BCG or HepB or IPV or DTP-containing or DTPCV or Td or DT or DTaP or Tdap or PCV or RV or MMR or MR or HPV or IIV or LAIV) adj3 vaccine*

[Outcome]
AND
Title, Abstract, Key words=
vaccination or immunization or immunisation or inoculation or uptake or coverage or rate* or accept* or hesitan* or access

[Study Design]
AND
Title, Abstract, Key words=
systematic review* or systematic literature review or systematic overview or meta analys* or metaanalys* or review

[Filters]
Publication date 2011-present

Annex S3: PRISMA-Flow template [60].

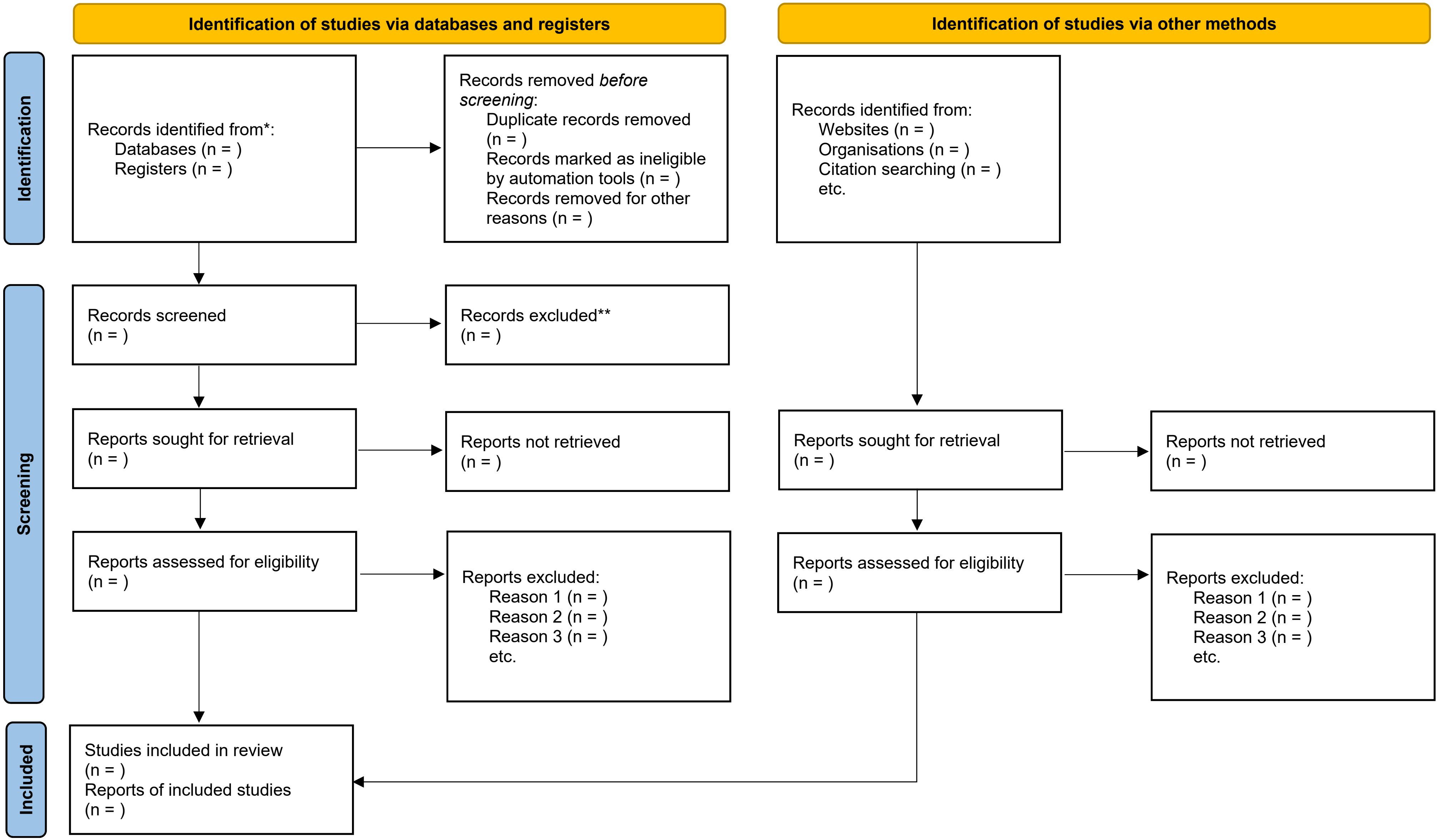

*Consider, if feasible to do so, reporting the number of records identified from each database or register searched (rather than the total number across all databases/registers).
**If automation tools were used, indicate how many records were excluded by a human and how many were excluded by automation tools.

Annex S4: Data extraction tables.

Author (Year)	Title (DOI)	Study Type	Included Studies (N)	Location/s	Vaccine/s	Measure of SE Inequality	Databases (Date)	Inclusion/Exclusion Criteria	Method of Synthesis	Main Findings	Mechanisms Identified	Risk of Bias
Name et al (0000)				Country 1 (N)<br>Country 2 (N)	Vaccine 1 (N)<br>Vaccine 2 (N)				Narrative<br>Meta-analysis	Effect estimates or key quotes.		
…												

Review Author (Year)	Primary Study Author (Year)	Title	Study Design
Name et al (0000)	Name et al (0000)		RCT<br>Non-RCT
…			

Annex S5: AMSTAR-2 checklist [61].

**1. Did the research questions and inclusion criteria for the review include the components of PICO?**		
For yes:<br>☐ Population<br>☐ Intervention<br>☐ Comparator group<br>☐ Outcome	Optional (recommended)<br>☐ timeframe for follow-up	☐ Yes<br>☐ No
**2. Did the report of the review contain an explicit statement that the review methods were established prior to the conduct of the review and did the report justify any significant deviations from the protocol?**		
For partial yes:<br>The authors state that they had a written protocol or guide that included ALL the following:<br>☐ review question(s)<br>☐ a search strategy<br>☐ inclusion/exclusion criteria<br>☐ a risk of bias assessment	For yes:<br>As for partial yes, plus the protocol should be registered and should also have specified:<br>☐ a meta-analysis/synthesis plan, if appropriate, *and*<br>☐ a plan for investigating causes of heterogeneity<br>☐ justification for any deviations from the protocol	☐ Yes<br>☐ Partial yes<br>☐ No
**3. Did the review authors explain their selection of the study designs for inclusion in the review?**		
For yes, the review should satisfy ONE of the following:<br>☐ *explanation for* including only RCTs<br>☐ OR *explanation for* including only NRSI<br>☐ OR *explanation for* including both RCTs and NRSI		☐ Yes<br>☐ No
**4. Did the review authors use a comprehensive literature search strategy?**		
For partial yes (all the following):<br>☐ searched at least 2 databases (relevant to research question)<br>☐ provided key words and/or search strategy<br>☐ justified publication restrictions (e.g. language)	For yes, should also have (all the following):<br>☐ searched the reference lists/bibliographies of included studies<br>☐ searched trial/study registries<br>☐ included/consulted content experts in the field<br>☐ where relevant, searched for grey literature<br>☐ conducted search within 24 months of completion of the review	☐ Yes<br>☐ Partial yes<br>☐ No
**5. Did the review authors perform study selection in duplicate?**		
☐ at least two reviewers independently agreed on selection of eligible studies and achieved consensus on which studies to include<br>☐ OR two reviewers selected a sample of eligible studies and achieved good agreement (at least 80 percent), with the remainder selected by one reviewer.		☐ Yes<br>☐ No

**6. Did the review authors perform data extraction in duplicate?**		
For yes, either ONE of the following:<br>☐ at least two reviewers achieved consensus on which data to extract from included studies<br>☐ OR two reviewers extracted data from a sample of eligible studies and achieved good agreement (at least 80 percent), with the remainder extracted by one reviewer.		☐ Yes<br>☐ No
**7. Did the review authors provide a list of excluded studies and justify the exclusions?**		
For partial yes:<br>☐ provided a list of all potentially relevant studies that were read in full-text form but excluded from the review	For yes, must also have:<br>☐ justified the exclusion from the review of each potentially relevant study	☐ Yes<br>☐ Partial yes<br>☐ No
**8. Did the review authors describe the included studies in adequate detail?**		
For partial yes (ALL the following):<br>☐ described populations<br>☐ described interventions<br>☐ described comparators<br>☐ described outcomes<br>☐ described research designs	For yes, should also have ALL the following:<br>☐ described population in detail<br>☐ described intervention in detail (including doses where relevant)<br>☐ described comparator in detail (including doses where relevant)<br>☐ described study's setting<br>☐ timeframe for follow-up	☐ Yes<br>☐ Partial yes<br>☐ No
**9. Did the review authors use a satisfactory technique for assessing the risk of bias (RoB) in individual studies that were included in the review?**		
**RCTs**<br>For partial yes, must have assessed RoB from:<br>☐ unconcealed allocation, *and*<br>☐ lack of blinding of patients and assessors when assessing outcomes (unnecessary for objective outcomes such as all-cause mortality)	For yes, must also have assessed RoB from:<br>☐ allocation sequence that was not truly random, and<br>☐ selection of the reported result from among multiple measurements or analyses of a specified outcome	☐ Yes<br>☐ Partial yes<br>☐ No<br>☐ Includes only NRSI
**NRSI**<br>For partial yes, must have assessed RoB from:<br>☐ confounding, *and*<br>☐ selection bias	For yes, must also have assessed RoB from:<br>☐ methods used to ascertain exposures, outcomes, *and*<br>☐ selection of the reported result from among multiple measurements or analyses of a specified outcome	☐ Yes<br>☐ Partial yes<br>☐ No<br>☐ Includes only RCTs
**10. Did the review authors report on the sources of funding for the studies included in the review?**		
For yes:<br>☐ must have reported on the sources of funding for individual studies included in the review. Note: Reporting that the reviewers looked for this information but it was not reported by study authors also qualifies		☐ Yes<br>☐ No

**11. If meta-analysis was performed did the review authors use appropriate methods for statistical combination of results?**

RCTs

For yes:
☐ the authors justified combining the data in a meta-analysis
☐ AND they used an appropriate weighted technique to combine study results and adjusted for heterogeneity if present
☐ AND investigated the causes of any heterogeneity

☐ Yes
☐ No
☐ No meta-analysis

NRSI

For yes:
☐ the authors justified combining the data in a meta-analysis
☐ AND they used an appropriate weighted technique to combine study results, adjusting for heterogeneity if present
☐ AND they statistically combined effect estimates from NRSI that were adjusted for confounding, rather than combining raw data, or justified combining raw data when adjusted effect estimates were not available
☐ AND they reported separate summary estimates for RCTs and NRSI separately when both were included in the review

☐ Yes
☐ No
☐ No meta-analysis

12. If meta-analysis was performed, did the review authors assess the potential impact of RoB in individual studies on the results of the meta-analysis or other evidence synthesis?

For yes:
☐ included only low risk of bias RCTs
☐ OR, if the pooled estimate was based on RCTs and/or NRSI at variable RoB, the authors performed analyses to investigate possible impact of RoB on summary estimates of effect

☐ Yes
☐ No
☐ No meta-analysis conducted

13. Did the review authors account for RoB in individual studies when interpreting/ discussing the results of the review?

For yes:
☐ included only low risk of bias RCTs
☐ OR, if RCTs with moderate or high RoB, or NRSI were included the review provided a discussion of the likely impact of RoB on the results

☐ Yes
☐ No

14. Did the review authors provide a satisfactory explanation for, and discussion of, any heterogeneity observed in the results of the review?

For yes:
☐ There was no significant heterogeneity in the results
☐ OR if heterogeneity was present the authors performed an investigation of sources of any heterogeneity in the results and discussed the impact of this on the results of the review

☐ Yes
☐ No

15. If they performed quantitative synthesis did the review authors carry out an adequate investigation of publication bias (small study bias) and discuss its likely impact on the results of the review?

For yes:
☐ performed graphical or statistical tests for publication bias and discussed the likelihood and magnitude of impact of publication bias

☐ Yes
☐ No
☐ No meta-analysis conducted

**16. Did the review authors report any potential sources of conflict of interest, including any funding they received for conducting the review?**	
For yes:	
☐ The authors reported no competing interests OR	☐ Yes
☐ The authors described their funding sources and how they managed potential conflicts of interest	☐ No

Annex S6: Citation matrix [63].

	Review 1	Review 2	...	Occurrence (N)
Primary Study 1				
Primary Study 2				
...				

Annex S7: Summary table.

Author (Year)	Location/s	Vaccine/s	Measure of SE Inequality	Key Findings	Mechanisms Identified	Type of Mechanism	AMSTAR 2 Rating
				Effect estimates<br>Key quotes			Low<br>Moderate<br>High

Annex S8: Pilot search 1 strategy.

	**Study Design**	
1	review.pt.	2987245
2	(medline or medlars or embase or pubmed or cochrane).tw,sh.	296414
3	(scisearch or psychinfo or psycinfo).tw,sh.	49246
4	(psychlit or psyclit).tw,sh.	914
5	cinahl.tw,sh.	37808
6	((hand adj2 search$) or (manual$ adj2 search$)).tw,sh.	15423
7	(electronic database$ or bibliographic database$ or computeri?ed database$ or online database$).tw,sh.	50629
8	(pooling or pooled or mantel haenszel).tw,sh.	132062
9	(peto or dersimonian or der simonian or fixed effect).tw,sh.	9513
10	(retraction of publication or retracted publication).pt.	23482
11	or/2-10	456683
12	1 and 11	194331
13	meta-analysis.pt.	165901
14	meta-analysis.sh.	165901
15	(meta-analys$ or meta analys$ or metaanalys$).tw,sh.	266288
16	(systematic$ adj5 review$).tw,sh.	279585
17	(systematic$ adj5 overview$).tw,sh.	3089
18	(quantitativ$ adj5 review$).tw,sh.	9665
19	(quantitativ$ adj5 overview$).tw,sh.	383
20	(quantitativ$ adj5 synthesis$).tw,sh.	3915
21	(methodologic$ adj5 review$).tw,sh.	7738
22	(methodologic$ adj5 overview$).tw,sh.	519
23	(integrative research review$ or research integration).tw.	162
24	or/13-23	424115
25	12 or 24	500272
	**Population**	
26	exp socioeconomic factors/ or sociodemographic factors/	494165

27	(socioeconomic or socio-economic or sociodemographic or sep or ses or class).mp.	838465
28	education/	21502
29	(education or lifelong learning or life-long learning or human capital or school* or literacy or academic achievement).mp.	1257312
30	work/	20242
31	(employ* or unemploy* or occupation* or job* or work or career* or vocation or economic activity or labour market activity or isco).mp.	2227277
32	(income or wealth or wage* or salar* or earning* or low-income or money).mp.	246861
33	((inequit* or inequalit* or unequal or equal* or equit* or depriv* or poverty or impoverished or disadvantage* or gradient or gap* or disparit* or difference*) adj3 economic).mp.	7627
34	26 or 27 or 28 or 29 or 30 or 31 or 32 or 33	4120827
	**Intervention**	
35	Injections/ or tuberculosis Vaccines/ or Hepatitis B Vaccines/ or Pertussis Vaccines/ or exp Diphtheria Toxoid/ or Tetanus Toxoid/ or Haemophilus Vaccines/ or exp poliovirus vaccines/ or exp Pneumococcal vaccines/ or rotavirus vaccines/ or exp measles vaccine/ or rubella vaccine/ or exp papillomavirus vaccines/ or influenza vaccines/ or exp COVID-19 vaccines	147148
36	(vaccine* or immunize or immunise or injection* or jab* or inoculate).mp.	1137349
37	((tb or tuberculosis or Hep B or Hepatitis B or diphtheria or tetanus or pertussis or whooping cough or hib or haemophilus or haemophilus influenzae type b or poliovirus or polio or poliomyelitis or pneumococcal or pneumococcus or rotavirus or measles or rubella or human papillomavirus or wart virus or influenza or flu or COVID-19 or COVID 19 or COVID19 or coronavirus or SARS-CoV-2 or SARS Cov 2 or severe acute respiratory syndrome) adj3 vaccine*).mp.	116029
38	((BCG or HepB or IPV or DTP-containing or DTPCV or Td or DT or DTaP or Tdap or PCV or PPV or RV or MMR or MR or HPV or IIV or LAIV) adj3 vaccine*).mp.	35403
39	35 or 36 or 37 or 38	1143673
40	25 AND 34 AND 39	2087

Annex S9: Pilot search 2 strategy.

	**Study Design**	
1	review.pt.	2987245
2	(medline or medlars or embase or pubmed or cochrane).tw,sh.	296414
3	(scisearch or psychinfo or psycinfo).tw,sh.	49246
4	(psychlit or psyclit).tw,sh.	914
5	cinahl.tw,sh.	37808
6	((hand adj2 search$) or (manual$ adj2 search$)).tw,sh.	15423
7	(electronic database$ or bibliographic database$ or computeri?ed database$ or online database$).tw,sh.	50629
8	(pooling or pooled or mantel haenszel).tw,sh.	132062
9	(peto or dersimonian or der simonian or fixed effect).tw,sh.	9513
10	(retraction of publication or retracted publication).pt.	23482
11	or/2-10	456683
12	1 and 11	194331
13	meta-analysis.pt.	165901
14	meta-analysis.sh.	165901
15	(meta-analys$ or meta analys$ or metaanalys$).tw,sh.	266288
16	(systematic$ adj5 review$).tw,sh.	279585
17	(systematic$ adj5 overview$).tw,sh.	3089
18	(quantitativ$ adj5 review$).tw,sh.	9665
19	(quantitativ$ adj5 overview$).tw,sh.	383
20	(quantitativ$ adj5 synthesis$).tw,sh.	3915
21	(methodologic$ adj5 review$).tw,sh.	7738
22	(methodologic$ adj5 overview$).tw,sh.	519
23	(integrative research review$ or research integration).tw.	162
24	or/13-23	424115
25	12 or 24	500272
	**Population**	
26	exp socioeconomic factors/ or sociodemographic factors/	494165

27	(socioeconomic or socio-economic or sociodemographic or sep or ses or class).mp.	838465
28	education/	21502
29	(education or lifelong learning or life-long learning or human capital or school* or literacy or academic achievement).mp.	1257312
30	work/	20242
31	(employ* or unemploy* or occupation* or job* or work or career* or vocation or economic activity or labour market activity or isco).mp.	2227277
32	(income or wealth or wage* or salar* or earning* or low-income or money).mp.	246861
33	((inequit* or inequalit* or unequal or equal* or equit* or depriv* or poverty or impoverished or disadvantage* or gradient or gap* or disparit* or difference*) adj3 economic).mp.	7627
34	26 or 27 or 28 or 29 or 30 or 31 or 32 or 33	4120827
	**Intervention**	
35	Injections/ or tuberculosis Vaccines/ or Hepatitis B Vaccines/ or Pertussis Vaccines/ or exp Diphtheria Toxoid/ or Tetanus Toxoid/ or Haemophilus Vaccines/ or exp poliovirus vaccines/ or exp Pneumococcal vaccines/ or rotavirus vaccines/ or exp measles vaccine/ or rubella vaccine/ or exp papillomavirus vaccines/ or influenza vaccines/ or exp COVID-19 vaccines	147148
36	(vaccine* or immunize or immunise or injection* or jab* or inoculate).mp.	1137349
37	((tb or tuberculosis or Hep B or Hepatitis B or diphtheria or tetanus or pertussis or whooping cough or hib or haemophilus or haemophilus influenzae type b or poliovirus or polio or poliomyelitis or pneumococcal or pneumococcus or rotavirus or measles or rubella or human papillomavirus or wart virus or influenza or flu or COVID-19 or COVID 19 or COVID19 or coronavirus or SARS-CoV-2 or SARS Cov 2 or severe acute respiratory syndrome) adj3 vaccine*).mp.	116029
38	((BCG or HepB or IPV or DTP-containing or DTPCV or Td or DT or DTaP or Tdap or PCV or PPV or RV or MMR or MR or HPV or IIV or LAIV) adj3 vaccine*).mp.	35403
39	35 or 36 or 37 or 38	1143673
40	25 AND 34 AND 39	2087
	**Outcome**	
41	exp vaccination/ or exp vaccination hesitancy/ or vaccination coverage/	102709
42	(vaccination or immunization or immunization or inoculation or uptake or coverage or rate* or accept* or hesitan* or access).mp.	4987546
43	41 or 42	4987546
44	40 AND 43	1282
45	limit 44 to yr=”2011 -Current”	1090

Annex S10: BMJ study design search filters [69].

1. review.pt.
2. (medline or medlars or embase or pubmed or cochrane).tw,sh.
3. (scisearch or psychinfo or psycinfo).tw,sh.
4. (psychlit or psyclit).tw,sh.
5. cinahl.tw,sh.
6. ((hand adj2 search$) or (manual$ adj2 search$)).tw,sh.
7. (electronic database$ or bibliographic database$ or computeri?ed database$ or online database$).tw,sh.
8. (pooling or pooled or mantel haenszel).tw,sh.
9. (peto or dersimonian or der simonian or fixed effect).tw,sh.
10. (retraction of publication or retracted publication).pt.
11. or/2-10
12. 1 and 11
13. meta-analysis.pt.
14. meta-analysis.sh.
15. (meta-analys$ or meta analys$ or metaanalys$).tw,sh.
16. (systematic$ adj5 review$).tw,sh.
17. (systematic$ adj5 overview$).tw,sh.
18. (quantitativ$ adj5 review$).tw,sh.
19. (quantitativ$ adj5 overview$).tw,sh.
20. (quantitativ$ adj5 synthesis$).tw,sh.
21. (methodologic$ adj5 review$).tw,sh.
22. (methodologic$ adj5 overview$).tw,sh.
23. (integrative research review$ or research integration).tw.
24. or/13-23
25. 12 or 24
